# Alternative epidemic indicators for COVID-19 in three settings with incomplete death registration systems

**DOI:** 10.1126/sciadv.adg7676

**Published:** 2023-06-09

**Authors:** Ruth McCabe, Charles Whittaker, Richard J. Sheppard, Nada Abdelmagid, Aljaile Ahmed, Israa Zain Alabdeen, Nicholas F. Brazeau, Abd Elhameed Ahmed Abd Elhameed, Abdulla Salem Bin-Ghouth, Arran Hamlet, Rahaf AbuKoura, Gregory Barnsley, James A. Hay, Mervat Alhaffar, Emilie Koum Besson, Semira Mitiku Saje, Binyam Girma Sisay, Seifu Hagos Gebreyesus, Adane Petros Sikamo, Aschalew Worku, Yakob Seman Ahmed, Damen Haile Mariam, Mitike Molla Sisay, Francesco Checchi, Maysoon Dahab, Bilal Shikur Endris, Azra C. Ghani, Patrick G. T. Walker, Christl A. Donnelly, Oliver J. Watson

**Affiliations:** ^1^ Department of Statistics, University of Oxford, Oxford, UK.; ^2^ NIHR Health Research Protection Unit in Emerging and Zoonotic Infections, Liverpool, UK.; ^3^ MRC Centre for Global Infectious Disease Analysis, Department of Infectious Disease Epidemiology, Imperial College London, London, UK.; ^4^ Department of Infectious Disease Epidemiology, Faculty of Epidemiology and Population Health, London School of Hygiene and Tropical Medicine, London, UK.; ^5^ Sudan COVID-19 Research Group, Khartoum, Sudan.; ^6^ Sudan Youth Peer Education Network, Khartoum, Sudan.; ^7^ University of North Carolina School of Medicine, Chapel Hill, NC, USA.; ^8^ Department of Community Medicine, Hadhramout University, Mukalla, Yemen.; ^9^ Center for Communicable Disease Dynamics, Harvard T. H. Chan School of Public Health, Boston, MA, USA.; ^10^ Syria Research Group (SyRG), co-hosted by the London School of Hygiene and Tropical Medicine, London, UK and Saw Swee Hock School of Public Health, Singapore, Singapore.; ^11^ School of Public Health, College of Health Sciences, Addis Ababa University, Addis Ababa, Ethiopia.; ^12^ School of Exercise and Nutrition Science, Institute for Physical Activity and Nutrition (IPAN), Deakin University, Melbourne, Victoria, Australia.; ^13^ School of Medicine, College of Health Sciences, Addis Ababa University, Addis Ababa, Ethiopia.; ^14^ Ethiopian Federal Ministry of Health, Addis Ababa, Ethiopia.

## Abstract

Not all COVID-19 deaths are officially reported, and particularly in low-income and humanitarian settings, the magnitude of reporting gaps remains sparsely characterized. Alternative data sources, including burial site worker reports, satellite imagery of cemeteries, and social media–conducted surveys of infection may offer solutions. By merging these data with independently conducted, representative serological studies within a mathematical modeling framework, we aim to better understand the range of underreporting using examples from three major cities: Addis Ababa (Ethiopia), Aden (Yemen), and Khartoum (Sudan) during 2020. We estimate that 69 to 100%, 0.8 to 8.0%, and 3.0 to 6.0% of COVID-19 deaths were reported in each setting, respectively. In future epidemics, and in settings where vital registration systems are limited, using multiple alternative data sources could provide critically needed, improved estimates of epidemic impact. However, ultimately, these systems are needed to ensure that, in contrast to COVID-19, the impact of future pandemics or other drivers of mortality is reported and understood worldwide.

## Teaser

Alternative data sources are valid tools to track epidemic dynamics and assess the under-ascertainment of COVID-19 mortality.

## INTRODUCTION

Accurate ascertainment of infections, cases, and deaths of an emerging infectious disease is vital to implement an effective public health response. However, these quantities depend on both testing capacity and robust vital registration systems. Consequently, only a subset of the true burden of a disease is captured within official statistics. During the coronavirus disease 2019 (COVID-19) pandemic, official COVID-19 data obscured the perception of the global burden of the disease (*1*). For example, in some settings with low reported COVID-19 death tolls, serological surveys (serosurveys) have revealed extensive severe acute respiratory syndrome coronavirus 2 (SARS-CoV-2) transmission (*2*–*4*). These levels of transmission are inconsistent with the number of infections expected based on officially reported deaths and estimates of the infection fatality ratio (IFR) (*5*, *6*), with the most parsimonious explanation that COVID-19 deaths go unreported (*7*).

Due to the underreporting of COVID-19 deaths (*1*), excess mortality has been used frequently as an alternative means by which to assess the impact of the COVID-19 pandemic (*8*). On 5 May 2022, the World Health Organization (WHO) published estimates of the “full” global death toll of the pandemic based on all-cause excess mortality (*9*). Although not subject to the same biases as COVID-19 deaths, which typically rely on a proven SARS-CoV-2 infection around the time of the death, estimates of excess mortality still require complete data on the total number of deaths from all causes, which in turn requires mechanisms by which to record these deaths. Unfortunately, robust vital registration systems do not exist in many parts of the world, with the WHO estimating in 2020 that 40% of the world’s deaths from all causes occur unregistered (*10*).

In settings without official all-cause mortality data, estimation of excess mortality during the pandemic has relied solely on model-based inference by pooling information from countries with similar socioeconomic and demographic characteristics. An alternative approach is to leverage alternative mortality data sources and epidemic indicators that can rapidly provide a more data-driven understanding of epidemic dynamics, such as social media–shared obituary notifications (*11*). Characterizing the potential biases in these innovative data sources is crucial to understanding their suitability for tracking the spread of SARS-CoV-2, as well as more broadly for mortality monitoring in the absence of complete vital registration. In response, we consider here three alternative data sources generated during the pandemic to understand the transmission of SARS-CoV-2 in two cities in sub-Saharan Africa and one in the Middle East. These include burial site worker reports in Addis Ababa (*12*), satellite imagery of cemeteries in Aden (*13*), and social media–conducted surveys of symptomatic infection in Khartoum (*14*) (Table 1).

**Table 1.** Sources of alternative data collected at study locations and seroprevalence estimates. See the Supplementary Materials for further details of each study and the specifics of assays used.

Setting	Alternative data source		Seroprevalence		
	Overview	Reference	Overview	Reported estimate	Reference
Addis Ababa, Ethiopia	Burial site worker cemetery reports 01/01/2015 to 26/01/2021	(*12*)	Random sample of 956 households 22/07/2020 to 10/08/2020	IgG: 1.9% (95% CI, 0.4–3.7%) Combined IgG/IgM: 3.5% (95% CI, 1.7–5.4%)	(*17*)
Aden, Yemen	Satellite imagery of cemetery burials 21/07/2016 to 19/09/2020	(*13*)	Cross-sectional household study of 2001 people 28/11/2020 to 13/12/2020	IgG: 25% (95% CI, 23.2–26.9%) IgM: 0.2% (95% CI, 0.1–0.4%) IgG & IgM: 2.3% (95% CI, 1.7–2.9%) Combined IgG/IgM: 27.4% (95% CI, 25.6–29.3%)	(*19*)
Khartoum, Sudan	Survey of historic symptomatic infections conducted through social media channels 26/05/2020 to 03/06/2020	(*14*)	Cross-sectional household study of 2375 people 01/03/2021 to 10/04/2021	Combined IgG/IgM: 54.6% (95% CI, 51.4–57.8%)	(*21*)

In each setting, representative serosurveys have also been conducted independently at different time points before the availability of vaccines. We incorporate these within a previously developed mathematical modeling framework (*11*) to quantify how informative each data source is for explaining population seroprevalence under different assumptions for the IFR in each setting. In brief, the model uses a Susceptible-Exposed-Infected-Recovered structure, which explicitly captures pathways through hospital care and the impact this has on mortality (see Materials and Methods for further information). Our default assumption is that the relationship between the IFR and age is consistent with the log-linear relationship estimated in high-income settings as estimated in multiple modeling studies (*5*, *6*) and meta-analyses (*15*), although there is evidence that IFR may be higher in lower-income settings (*7*). We test this hypothesis first by fitting to the alternative sources for mortality (Addis Ababa and Aden) and case incidence (Khartoum) data using the relationship between IFR and age as estimated by Brazeau *et al.* (*6*). From these model fits, we compare the model-predicted seroprevalence against the reported seroprevalence estimates using a chi-square test, arguing that this is good evidence that the alternative datasets are reliable for tracking the transmission of SARS-CoV-2 and the assumed IFR is suitable should these values not differ significantly. In addition, in settings where the alternative sources and the serosurveys do not agree, it presents an opportunity to better understand the biases in these alternative datasets and explore alternative hypotheses for the severity of COVID-19 in these settings. Full methods are presented in Materials and Methods and in the Supplementary Materials.

## RESULTS

### Addis Ababa

In Addis Ababa, excess mortality was derived from cemetery surveillance data collected via the Addis Ababa Mortality Surveillance Program across January 2015 to January 2021, as detailed by Endris *et al.* (*12*). These data comprise the number of burials per day at all cemeteries in the city according to official records kept by workers at each site. As cremation is not practiced in Addis Ababa, this surveillance program is expected to capture the vast majority of deaths from all causes in the city (*12*).

**Fig. 1.**
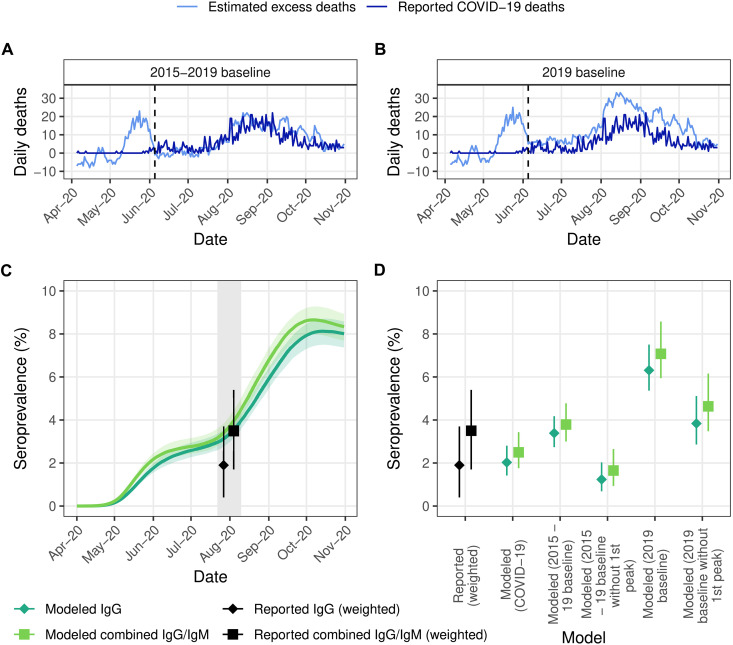
Mortality reporting and seroprevalence in Addis Ababa, Ethiopia in 2020. (**A**) Reported COVID-19 deaths compiled from the Ethiopian Public Health Institute and estimated excess mortality using cemetery surveillance from (*12*) using data from 2015 to 2019 to derive the baseline. Dashed line indicates the end of the early peak in excess mortality featured in the sensitivity analysis. (**B**) As in (A), but with excess mortality derived from 2019 data only. (**C**) Estimated seroprevalence from model fit to excess mortality under 2015–2019 baseline (median and 95% credible intervals) with the first peak compared to reported values by Abdella *et al.* (*17*). Gray shaded area highlights the sampling period of the serosurvey, with weighted reported estimates both corresponding to this entire period. (**D**) Seroprevalence estimated under models fit to either reported COVID-19 or different estimates of excess mortality with different baselines (median and 95% credible intervals) compared to reported seroprevalence in (*17*).

Reported COVID-19 deaths (*16*) largely matched estimated excess mortality from June 2020 onward, although this was in part dependent on how baseline mortality was estimated (Fig. 1, A and B). While the annual number of burials in each year from 2015 to 2018 was largely consistent (average, 12,862; SD, 389), there was a significant reduction in the number of burials observed in 2019 compared to the mean from 2015 to 2018 (total, 11,256; χ^2^ test, *P* < 0.001; fig. S3). Therefore, we explored two methods for estimating baseline mortality in 2020, in which we used mortality data either from 2015 to 2019 or exclusively from 2019 [Spearman correlation between excess mortality and COVID-19 deaths over our study period: *r* = 0.59 (using 2015–2019 as baseline) and *r* = 0.69 (using just 2019 as baseline)]. Regardless of the baseline, we observed a peak in excess mortality observed in May 2020 that was not associated with a peak in reported COVID-19 deaths (Fig. 1, A and B). We explored whether this peak could be due to COVID-19 through an additional sensitivity analysis by starting the model from 5 June 2020 instead of 6 April 2020 (the date of the first COVID-19 death; Fig. 1D) (Spearman correlation between excess mortality and COVID-19 deaths from June 2020 onward: *r* = 0.74 and *r* = 0.75 for 2015–2019 and 2019-only baselines, respectively). Therefore, we considered four excess mortality scenarios in total for Addis Ababa, which we assumed to be equally plausible a priori.

We fitted the mathematical model separately to reported COVID-19 deaths and the four estimated excess mortality time series and compared the number of infections estimated by the model to those at the time of a serosurvey conducted by Abdella *et al.* (*17*) in July to August 2020. The study used random sampling of 956 households in the city and reported a weighted prevalence of immunoglobulin G (IgG) and combined IgG/IgM antibodies of 1.9% (0.4 to 3.7%) and 3.5% (1.7 to 5.4%), respectively, having post-stratified by age and sex to account for potential biases in their sample (see the Supplementary Materials for further information).

The model fit to COVID-19 deaths produced estimates of seroprevalence not significantly different from the values reported by Abdella *et al.* (*17*) (*P*: 0.884 and 0.334 for IgG and combined IgG/IgM, respectively), suggesting that officially reported deaths are representative of the true COVID-19 death toll in this setting. Similarly, we observed that most of the seroprevalence estimates based on model fits to the excess mortality inferred from cemetery burial data were not statistically significantly different from the reported seroprevalence by Abdella *et al.* (*17*) for both antibody types (table S2). However, this is expected given the similarity in the COVID-19 and excess mortality time series (Fig. 1, A and B). The exception to this was the model fit to excess mortality inferred using 2019 only as the baseline and including the first peak (*P*: <0.001 and 0.002 for IgG and combined IgG/IgM antibodies, respectively), with the resulting model-estimated seroprevalence three times and twice as great as the reported prevalence of IgG and combined IgG/IgM antibodies of Abdella *et al.* (*17*) (Fig. 1D). This suggests that excess mortality under this baseline scenario overestimated COVID-19 mortality and is unsuitable for informing the size of the COVID-19 epidemic. Consequently, we approximate that, depending on the selection of mortality baseline, between 68.7% (1064 of 1549, reflecting the 2015–2019 scenario with the first peak in May included) and 100% of COVID-19 deaths were reported across April to November 2020 in Addis Ababa. The upper estimate of 100% (complete) reporting reflects both the near agreement between the cemetery-inferred excess mortality and reported COVID-19 deaths, and the nonsignificant difference between the seroprevalence inferred on the basis of model fits to reported COVID-19 deaths and the reported seroprevalence (Fig. 1C and table S5).

### Aden

**Fig. 2.**
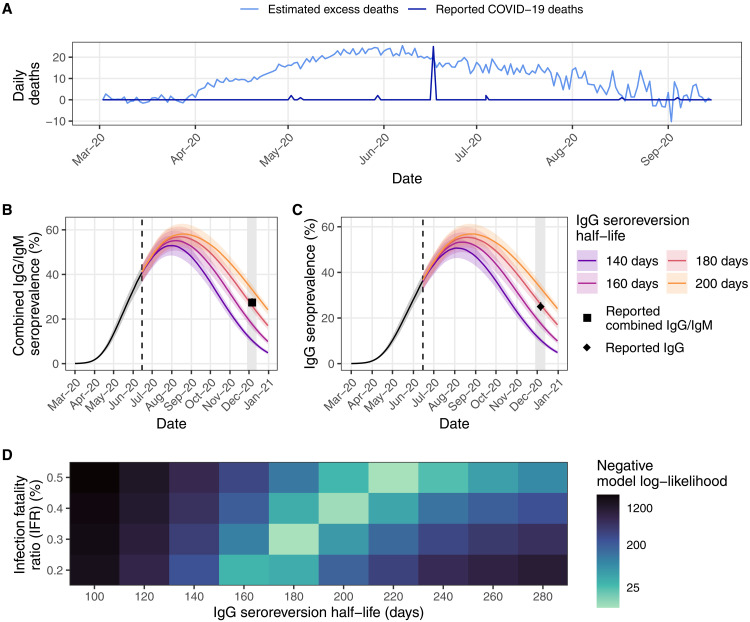
Mortality reporting and seroprevalence in Aden, Yemen in 2020. (**A**) Reported COVID-19 deaths and estimated excess mortality from satellite surveillance of burials from (*13*). (**B**) Estimated seroprevalence of combined IgG/IgM (positive for either IgG and/or IgM) from the model fitted to excess mortality using the default IFR (= 0.3%) under different IgG seroreversion half-lives compared to observed values from (*19*) [reported seroprevalence: 27.4% (95% CI, 25.6 to 29.3%)]. Dashed vertical line indicates point in time when seroprevalence dynamics divert due to difference in IgG half-life modeled. (**C**) As in (B) but for IgG antibodies [reported seroprevalence: 25.0% (95% CI, 23.2 to 26.9%)]. (**D**) Combined negative log-likelihood of models of IgG and combined IgG/IgM antibodies under varying assumptions of the IFR and IgG seroreversion half-life. In (B) and (D), the IgM seroreversion half-life is held constant at 50 days. Values associated with the highest log-likelihoods (shown here in light blue) indicate the best fit of the parameter values to the observed data.

In Aden, excess mortality was estimated from satellite imagery surveillance of all cemeteries in the city to estimate daily burials between July 2016 and September 2020, by quantifying expansions to cemetery surface area and validating this with civil death registrations (*13*). From these data, a wave of excess mortality was estimated to have occurred between April and September 2020, peaking in early June. Reported COVID-19 deaths were only available via scraping death counts tweeted daily by the Yemen Supreme National Emergency Committee for COVID-19 (*18*). Excess mortality estimates suggested that 2120 [95% confidence interval (CI), 424 to 4137] excess deaths occurred across 1 April to 19 September 2020, compared to just 34 officially reported COVID-19 deaths during this same period (*18*), implying substantial under-ascertainment of COVID-19 mortality (Fig. 2A).

In December 2020, Bin-Ghouth *et al.* (*19*) conducted a cross-sectional household serosurvey of 2001 people and estimated that 25.0% (95% CI, 23.2 to 26.9%) and 0.2% (95% CI, 0.1 to 0.4%) of the population had either IgG or IgM antibodies to SARS-CoV-2, respectively, with a total of 27.4% (95% CI, 25.6 to 29.3%) estimated to have IgG and/or IgM antibodies (combined IgG/IgM). Because of the long delay of approximately 5 months between the peak in excess mortality and the implementation of the serosurvey in this setting, it was necessary to consider the decay of antibodies after infection over time. Antibody kinetics vary substantially among individuals, resulting in high uncertainty around the duration of seropositivity after infection (*20*). To the best of our knowledge, these parameters had not been quantified for the assay used in this study, and so we conducted sensitivity analyses to explore the impact of different assumptions for IgG and IgM seroreversion. IgM antibodies endure for a substantially shorter duration than IgG antibodies. Hence, we found that varying the IgM half-life had a minimal impact in the context of this analysis and thus assumed a central value of 50 days as in (*6*) (nonstatistically significant differences with reported values; fig. S11 and table S3), but that the IgG half-life was an important determinant of our estimated seroprevalence (Fig. 2 and table S3).

Seroprevalence inferred from the model fit to reported COVID-19 deaths was statistically significantly different from that reported for all antibody types and under all seroreversion half-lives considered (*P*: <0.001 for IgG and combined IgG/IgM, <0.009 for IgM), suggesting that official reported COVID-19 deaths substantially underestimate the true death toll. However, in model fits to the satellite-derived excess mortality, we found no statistically significant differences between reported and modeled seroprevalence of IgG and combined IgG/IgM antibodies (*P*: 0.651 and 0.563, respectively) with an assumed IgG seroreversion half-life of 180 days. This finding suggests that these data are a more accurate representation of the first COVID-19 outbreak in Aden than officially reported deaths.

Although the default IFR (IFR = 0.3%) and IgG seroreversion half-life (180 days) maximized the combined model log-likelihood of IgG and combined IgG/IgM antibodies, we were able to find other parameter combinations that could also explain the reported seroprevalence (Fig. 2). We found that a slightly higher IFR of 0.4 or 0.5% combined with a slightly longer seroreversion half-life of 200 or 220 days, respectively, also produced nonstatistically significant differences between reported and modeled seroprevalence of both antibody types (*P*: 0.289 and 0.649 for IgG and 0.944 and 0.385 for combined IgG/IgM antibodies, respectively) and produced similarly high log-likelihoods (Fig. 2 and table S3). Although a lower IFR of 0.2% was able to also produce nonstatistically significant differences when using shorter seroreversion half-lives (table S3), the model was unable to recreate the excess deaths from the satellite data, suggesting that IFR in Aden was greater than 0.2% (fig. S10).

Overall, the broad agreement between the two independent data sources under plausible assumptions of antibody seroreversion and the IFR strongly supports COVID-19 to be the driver of the observed satellite-inferred excess mortality. Consequently, we estimate that just 1.6% (34 of 2120) of COVID-19 deaths in Aden in summer 2020 were captured in official statistics (table S5). On the basis of the uncertainty in the satellite-inferred excess mortality (95% CI, 424 to 4,137), we report our uncertainty in the reporting fraction to be between 0.8 and 8.0%.

### Khartoum

In Khartoum, we leveraged an online survey distributed through social media channels (*14*). The survey presented respondents with a list of symptoms known to be associated with SARS-CoV-2 infection and used to triage patients in Sudan. Respondents were asked which of these symptoms they had experienced since the start of the pandemic. Respondents were also asked both whether they had received (i) a SARS-CoV-2 diagnostic test and (ii) the outcome of any test taken. From this survey, the SARS-CoV-2 infection status of survey participants who had not received a COVID-19 test was inferred based on their reported symptom(s), resulting in an estimate of the symptomatic attack rate in the general population. The survey collected responses opportunistically, resulting in 5018 responses from individuals over 15 years old throughout Khartoum State between 26 May and 3 June 2020. From these respondents, 11.0% of individuals were estimated to have experienced a symptomatic infection by 3 June, with lower and upper estimates (based on different methods for inferring symptomatic infection) of 8.3 and 13.7%.

**Fig. 3.**
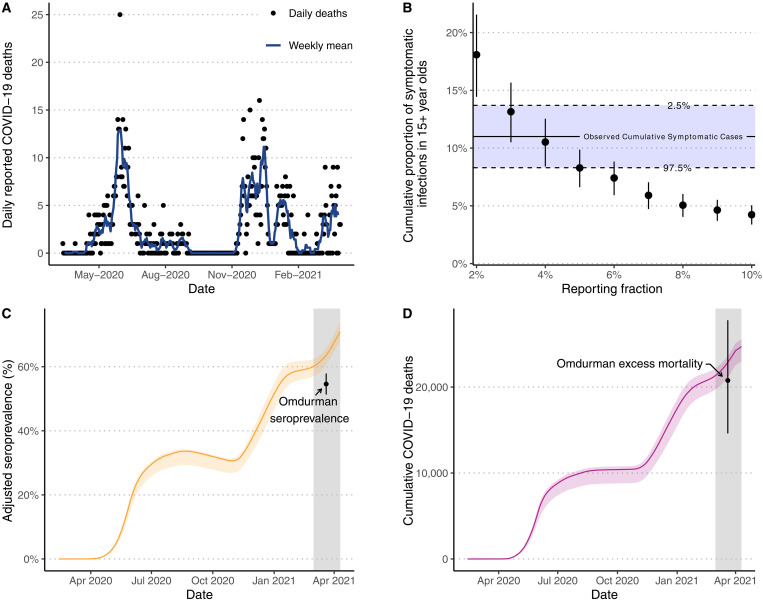
Estimates of under-ascertainment of deaths in Khartoum. (**A**) Daily and weekly mean reported COVID-19 deaths in Khartoum. We fit models to the reported COVID-19 deaths in (A) under different assumptions for what proportion of the true number of COVID-19 deaths these represent (reporting fractions). In (**B**), the resultant model fits were used to estimate the proportion of individuals aged over 15 years that would have experienced a symptomatic COVID-19 infection by 3 June 2020. The points and vertical bars show the median and 95% CI for each model fit and are compared against the observed cumulative number of symptomatic cases in Khartoum estimated from a social media–distributed survey (*14*), suggesting that 4% of COVID-19 deaths were detected. A cross-sectional household mortality and serosurvey conducted in Omdurman in March to April 2021 estimated seroprevalence to be 54.6% (95% CI, 51.4 to 57.8%) after adjusting for specific test performance (*21*). This estimate is depicted in (**C**) by point and whiskers and is compared against the adjusted seroprevalence (median and 95% CI shown in orange) predicted by a model fit with an assumed mortality reporting fraction of 4%. On the basis of the same survey, 20,766 (95% CI, 14,641 to 27,750) excess deaths are estimated to have occurred across Khartoum state. This estimate is depicted in (**D**) by point and whiskers and is compared against the cumulative number of COVID-19 deaths (median and 95% CI shown in purple) predicted by a model fit with an assumed mortality reporting fraction of 4%. In (C) and (D), the gray shaded area highlights the sampling period of the serosurvey and mortality survey in Omdurman.

By the end of March 2021 (before a pause in publication of COVID-19 reports by the Sudan Federal Ministry of Health), 938 COVID-19 deaths had been reported in Khartoum (Fig. 3A). We fit the transmission model to the observed COVID-19 deaths, making the assumption that the reported COVID-19 deaths reflected a fixed proportion of the true total number of COVID-19 deaths over time (figs. S12 and S13). From these, our central estimate is that 4% of COVID-19 deaths were reported based on comparison against the 11.0% of individuals estimated to have experienced a symptomatic infection by 3 June (Fig. 3B). We report our uncertainty in the reporting fraction to be between 3 and 6%, with the modeled 95% CI for the cumulative proportion of symptomatic infections overlapping with the lower or upper bounds reported of 8.3 and 13.7% (Fig. 3B).

Between 1 March and 10 April 2021, a cross-sectional household mortality and seroprevalence survey was conducted in Omdurman—a city in Khartoum state estimated to encompass ~35% of the population of Khartoum state (*21*). In this survey, 54.6% (95% CI, 51.4 to 57.8%) of the population were seropositive and 7113 excess deaths (95% CI, 5015 to 9505) were estimated to have occurred, which when scaled linearly to reflect the population of Khartoum state would be 20,766 (95% CI, 14,641 to 27,750) excess deaths (*21*). By comparison, with 4% of COVID-19 deaths assumed to have been detected, we estimate that 63.6% (95% CI, 60.6 to 65.7%) of the population would have been seropositive by 20 March 2021 (Fig. 3C), which is significantly different to the reported seroprevalence of 54.6% (χ^2^ test, *P* < 0.001; table S4), and that 22,870 (95% CI, 21,150 to 23,640) deaths would have occurred (Fig. 3D). Consequently, while the inferred reporting fraction of 4% based on the symptomatic survey yields estimates of the epidemic size that are similar to those observed in Omdurman almost a year later, estimates of the epidemic size are most accurately recreated with an assumed reporting fraction of 4.5% (fig. S14 and table S5).

## DISCUSSION

In this study, we demonstrate the validity of using alternative data sources, namely, burial site worker reports in Addis Ababa (*12*), satellite imagery of cemeteries in Aden (*13*), and social media–conducted surveys of symptomatic infection in Khartoum (*14*), to track the dynamics and burden of the COVID-19 epidemic in each of these settings. By incorporating these data within a previously published mathematical modeling framework (*11*), we could infer the number of infections implied by each source and validate the accuracy of these results by comparing them with independently conducted serosurveys. Consequently, we estimate that between 67 and 100% of COVID-19 deaths were reported in Addis Ababa, while in Aden and Khartoum, we demonstrated that there are likely to have been large unobserved epidemics with 0.8 to 8.0% and 3.0 to 6.0% of deaths reported, respectively (table S5).

Understanding the impact and burden of an infectious disease is integral to facilitating an effective public health response. Our results challenge the perception of the global burden of the disease as reported in official figures which, as shown here in the cases of Aden and Khartoum, can suffer from underreporting in resource-poor settings. In each setting, we found nonstatistically significant differences between our model-estimated seroprevalence based on the alternative data source and those reported in serosurvey, each of which tested at least 950 participants. This provides reasonable confidence in the accuracy of these sources to describe and understand the respective epidemic dynamics (tables S2 to S4). Hence, we consider that these alternative data sources represent a viable, and likely cost-effective, route to estimating disease burden and impact in settings where high-quality civil registration system mortality data are not currently available.

Our framework uses the IFR when comparing mortality inferred from alternative data sources to reported seroprevalence. Although this parameter has not been estimated directly within the three settings in this study, we have demonstrated that the default IFR accounting for the demography in Addis Ababa (IFR = 0.22%) estimated by Brazeau *et al.* (*6*) accurately captured the reported seroprevalence. However, in Aden, our results suggested that the IFR is at least 0.3%, which is higher than the IFR estimated by Brazeau *et al.* (*6*) after accounting for the demography in Aden (IFR = 0.17%). Similarly, in Khartoum, an IFR of approximately 0.38% accurately captured the reported seroprevalence, which is notably higher than the default IFR estimated by Brazeau *et al.* (*6*) after accounting for the demography in Khartoum (IFR = 0.20%). These studies should not be viewed as an accurate assessment of the IFR, with the mortality inferred from these alternative data unlikely to perfectly reflect the true COVID-19 mortality in each setting. Nonetheless, in addition to demonstrating that these data are indeed informative for describing and understanding epidemic dynamics, our results provide further evidence that the relationship between age and IFR that has been consistently observed in high-income countries (*5*, *6*) also exists in these low-income settings. It is further possible that the aforementioned relationship with age could lead to an underestimation of the IFR in some settings, as suggested by a recent meta-analysis of data from developing countries (*7*) and supported by our presented analyses of data from Aden and Khartoum.

Our results could be further corroborated via sources other than those directly used in our analyses. In Aden, multiple reports of hospital capacity being reached coincided with the epidemic peak estimated via satellite image–derived excess mortality (*13*, *22*). The saturation of healthcare facilities could also explain the higher IFR indicated in this setting. A similar situation may also have occurred in Khartoum, with over 70% of health centers closed in Khartoum as a COVID-19 containment measure during the first wave (*23*). Additional serosurveys have also been conducted in each setting. In Khartoum, a seroprevalence survey conducted in 22 neighborhoods and relying on voluntary enrolment organized through resistance committees estimated a seroprevalence of 18.3% (95% CI, 16.0 to 20.9%) using rapid antibody immunochromatography tests between 22 May and 5 July 2020 (*24*). Polymerase chain reaction (PCR)–confirmed prevalence of infection in the same survey was estimated at 35.0% (95% CI, 32.1 to 38.0%), raising concerns that the sampling scheme had resulted in an upward bias. However, the seroprevalence inferred from our model fits with an assumed reporting fraction of 4% was consistent with this survey (*P* = 0.493), confirming previous viral kinetics modeling of this study (*25*). In Addis Ababa, higher seroprevalence of combined IgG/IgM antibodies has been estimated in other surveys (*26*), which estimated a value of 10.9% in August 2020, with this value rising to 53.7% in December 2020. However, these estimates were derived from sampling focused on health care workers, who are known to have a higher risk of exposure to SARS-CoV-2 than the general population (*27*). Similarly, samples collected from Médecins Sans Frontières staff in Aden between September and November 2020 and tested using rapid serology lateral flow tests resulted in an estimated seroprevalence of 19.4% (95% CI, 17.9 to 20.8%) (*28*). However, a follow-up survey of Médecins Sans Frontières staff in Aden during January 2021 [after a period with very few COVID-19 cases reported in Aden (*18*)] but analyzed using an electrochemiluminescence immunoassay resulted in an estimated seroprevalence of 59.0% (95% CI, 52.2 to 65.9%) (*28*). This study both shows high exposure in health care staff and demonstrates the importance of accounting for waning rapid test sensitivity. Last, excess mortality published by the WHO (*9*) yielded similar magnitudes to those estimated by our analyses in Aden and Khartoum. However, we estimate a much greater reporting fraction in Addis Ababa than the corresponding national estimate (tables S5 and S6 and fig. S15), highlighting how death registration measured in individual cities or locations often will not reflect the realities of the whole countries, especially during periods of crises.

There are a number of limitations of this analysis. First, our estimated reporting fractions correspond to the entire study period and do not account for time-varying trends in detection of COVID-19 deaths. Second, there is uncertainty as to whether all excess deaths can be attributed directly due to COVID-19 (*9*, *29*, *30*), which is why we have conducted extensive sensitivity analyses to assess the robustness of our analysis in all three settings. Third, while we have demonstrated strong agreement between the alternative sources and seroprevalence estimates, it is still possible that the mortality inferred from the alternative source does not capture all deaths in each setting. For example, the Global Burden of Disease (GBD) (*31*) model suggests higher annual mortality in Addis Ababa than that presented in the cemetery surveillance data used in this study. While the GBD is a model-based estimate and the Addis Ababa Mortality Surveillance Program has shown to be representative of historic censuses (*32*), it is still likely that at least some deaths are missed by the cemetery data. If the true excess death toll in Addis Ababa is much higher than found in the Addis Ababa Mortality Surveillance Program, this would suggest that the IFR in Addis Ababa would need to be higher than in this analysis to be in agreement with the observed seroprevalence. Fourth, SeroTracker (*33*) suggested that all three serosurveys may be subject to “moderate” bias, with corresponding estimates defined as “likely correct for the target population”, as opposed to “very likely correct” for studies with “low” bias, based on nine criteria including sampling technique, sample size, and consistency of reporting results (*33*, *34*). Nonetheless, throughout our study, we have considered the full range of uncertainty estimated in the serosurveys and also estimated from our model fits, with these intervals shown to overlap under certain assumptions in each study. Analogously to the mortality data, we have focused on comparing the range of uncertainty intervals as opposed to point estimates alone. This is also applicable to the social media–based survey in Khartoum, which may suffer from self-reporting biases. Fifth, SARS-CoV-2 assays are often developed on high-income populations and the applicability to other settings has been questioned (*35*), which could influence the results. Sixth, we were unable to obtain specific seroconversion and seroreversion rate estimates for the assays used in these studies and had to rely on a sensitivity analysis around estimates derived from studies of other assays. Similarly, our modeling framework operates under the assumption of independence between the likelihood of the detection of IgG and combined IgG/IgM antibodies due to a lack of data to prove otherwise. These assumptions may explain why we were unable to recreate the relatively large difference between IgG and combined IgG/IgM antibodies observed in the serosurvey in Addis Ababa, despite adjusting for different seroreversion half-lives of these two antibody measurements. Crucially, our qualitative conclusions about the substantial degree of underreporting and the usefulness of these alternative data sources are robust to the limitations described above. Consequently, while there are likely biases in the alternative data sources leveraged, we argue that these biases are substantially smaller than those in official reported COVID-19 statistics and are thus vitally useful as proxies for COVID-19 mortality and epidemic dynamics.

The COVID-19 pandemic has caused a substantial loss of life, but reported deaths are likely to only capture a small proportion of the true death toll in settings without robust vital registration. In the absence of all-cause mortality data, we have validated the suitability of alternative data sources, namely, burial site worker reports, satellite imagery of cemeteries, and social media–conducted surveys of symptomatic infection, in tracking epidemics in Addis Ababa, Aden, and Khartoum across 2020. These sources provide a critical insight into the dynamics of SARS-CoV-2 in these settings and contradict the hypothesis that low-income countries were spared the worst of the pandemic. Our flexible, data-driven modeling framework is readily adaptable to other settings in which there are alternative sources of mortality and estimates of seroprevalence, such as Lusaka (*36*) and Haiti (*37*). However, while the modeling framework can be adapted to other settings and further alternative data sources, not all data sources will be suitable in other locations. For example, cemetery burial data are unlikely to be representative in settings that practice cremation and satellite imagery approaches may be less reliable in settings with extensive cloud coverage.

The global community must prioritize providing support and investment in the development of vital registration systems to capture all-cause mortality across the globe. Better vital registration is not only essential for future pandemic preparedness plans but also foundational for evaluating progress made across multiple areas of public health and is essential for the Sustainable Development Goals’ mission to “leave no one behind” (*38*).

## MATERIALS AND METHODS

We explain the general methodology, applicable to each of the three settings. Information on the specific datasets and sources for each modeled city are presented in the Supplementary Materials. A schematic diagram of the methods is shown in fig. S1. All data and code are provided at https://github.com/mrc-ide/covid-alternative-mortality/ (*39*).

### Mathematical model of SARS-CoV-2 transmission and disease progression

We use a previously published age-structured SARS-CoV-2 transmission model (*40*) and fitting framework (*11*) to fit the weekly estimated excess deaths in each setting in our study. In overview, the model is a Susceptible-Exposed-Infected-Recovered-Susceptible compartmental model, which is population-based and age-structured. The model explicitly represents disease severity and resultant passage through different health care levels, with an assumed elevated severity when health care capacity is exceeded as defined by Walker *et al.* (*40*). The model is capable of modeling vaccinations [see (*41*)], but in all settings considered in this study, vaccination campaigns had not started.

Model fitting was carried out within a Bayesian framework using a Metropolis-Hastings Markov chain Monte Carlo–based sampling scheme, which estimates the epidemic start date, *R*_0_, and the time varying reproduction number, *R_t_*, using a series of pseudo-random walk parameters (ρ*_n_*), which alter transmission every 2 weeks, given by

$$R_{t}=R_{0}.f(-\rho_{1}-\rho_{2}\cdots -\rho_{n})$$

where *f*(*x*) = 2. exp (*x*)/(1 + exp (*x*)). Each random walk parameter is introduced 2 weeks after the previous parameter, serving to capture changes in transmission every 2 weeks. The last change in transmission, *n*, is maintained for the last 4 weeks before the current day to reflect our inability to estimate the effect size of this parameter due to the approximate 21-day delay between infection and death. Each model fit is tailored to each setting, incorporating demographics, with the population size in 5-year age bands, and the effective number of general hospital beds and intensive care beds for each city.

### Estimation of seroprevalence

Seroprevalence over time was derived from the total number of infections estimated by the model, adjusted for rates of seroconversion, seroreversion, and serological assay sensitivity.

In each setting, we assumed that seroconversion followed an exponential distribution with mean time to seroconversion of 13.3 days for IgG antibodies (*42*). Seroconversion for IgM antibodies was assumed to occur more quickly, with mean time to seroconversion of 12.3 days (*42*). Similarly, in each setting, seroreversion was assumed to follow a Weibull distribution with shape parameter 3.7 (*6*) and with the scale parameter adjusted to enforce a specific half-life for IgG or IgM antibodies, respectively. As default, we assumed a half-life of 50 and 140 days for IgM and IgG, respectively, as estimated by Brazeau *et al.* (*6*). In Addis Ababa, a negligible amount of time had passed between the start of the epidemic inferred from the alternative mortality source and the serosurvey period, and consequently, the assumed duration of seroreversion does not affect our findings. However, because in Aden there was a substantial amount of time between the excess mortality and seroprevalence studies, we instead conducted a sensitivity analysis by varying the half-life of IgG antibodies between 100 and 280 days and of IgM antibodies between 30 and 70 days to capture the uncertainty surrounding the true value of these parameters. Last, in Khartoum, the observed seroprevalence estimated by Moser *et al.* (*21*) for Omdurman has already been adjusted to account for decreasing diagnostic test sensitivity over time as a result of waning antibody titers, which resulted in an increase from 34.3% crude seroprevalence to an adjusted seroprevalence of 54.6% (*21*). Consequently, we estimate seroprevalence from our model using a different approach. We continue to model the lag from infection to seroconversion, with a mean of 13.3 days. However, we consider individuals to only become seronegative again once they have moved from the recovered infection compartment to the susceptible compartment (i.e., by definition, they should not have antibodies to detect), which has a mean duration of 365 days and is described by an Erlang-2 distribution, i.e., is the sum of two independent exponential distributions each with a mean duration of 365/2 days.

Seroreversion and seroconversion distributions for combined IgG and IgM antibodies were estimated by treating the IgG and IgM distributions as independent. Although this assumption is unlikely to hold in reality, we validated our approach using Monte Carlo simulation, which produced an essentially identical probability distribution of seropositivity when the two antibody types were dependent as to that when they were treated independently (fig. S2).

### Testing for differences between observed and expected seroprevalence

Chi-square tests were used to determine whether the difference between observed and expected seroprevalence was statistically significant. Let θ_1_ denote reported seroprevalence and let θ_2 _denote our modeled seroprevalence. Our test statistic is

$$T=\frac{{\left(\theta_{1}-\theta_{2}\right)}^{2}}{\mathrm{var}(\theta_{1})+\mathrm{var}(\theta_{2})}$$

where $\mathrm{var}(\theta_{1})\approx {\left(\frac{\text{upper confidence limit}-\text{lower confidence limit}}{2\times 1.96}\right)}^{2}$, with 95% CI limits those reported in the serosurveys.

Under the null hypothesis *T*~χ_1_^2^, where χ_1_^2^ denotes a chi-square distribution with 1 degree of freedom, we assume that there are no statistically significant differences between θ_1 _and θ_2_. A *P* value greater than 0.05 indicates that there is not enough evidence to reject the null hypothesis, and we conclude that there are no statistically significant differences between the reported and modeled values.

### Estimation of reporting fractions

Reporting fractions are defined as total COVID-19 deaths over the period divided by total positive excess deaths over the same period (table S5).

## Supplementary Materials

### This PDF file includes:

Supplementary Materials and Methods Figs. S1 to S15 Tables S1 to S6 References
